# Design for learning – a case study of blended learning in a science unit

**DOI:** 10.12688/f1000research.7032.2

**Published:** 2015-11-16

**Authors:** Roslyn Gleadow, Barbara Macfarlan, Melissa Honeydew

**Affiliations:** 1 School of Biological Sciences, Monash University, Clayton, Victoria, 3088, Australia; 2 Office of the Vice-Provost (Learning and Teaching), Faculty of Science, Monash University, Clayton, Victoria, 3088, Australia; 3 School of Biochemistry and Molecular Biology, Monash University, Clayton, Victoria, 3088, Australia

**Keywords:** Blended learning, Moodle, educational design, science communication, on-line learning, higher education

## Abstract

Making material available through learning management systems is standard practice in most universities, but this is generally seen as an adjunct to the ‘real’ teaching, that takes place in face-to-face classes. Lecture attendance is poor, and it is becoming increasingly difficult to engage students, both in the material being taught and campus life. This paper describes the redevelopment of a large course in scientific practice and communication that is compulsory for all science students studying at our Melbourne and Malaysian campuses, or by distance education. Working with an educational designer, a blended learning methodology was developed, converting the environment provided by the learning management system into a teaching space, rather than a filing system. To ensure focus, topics are clustered into themes with a ‘question of the week’, a pre-class stimulus and follow up activities. The content of the course did not change, but by restructuring the delivery using educationally relevant design techniques, the content was contextualised resulting in an integrated learning experience. Students are more engaged intellectually, and lecture attendance has improved. The approach we describe here is a simple and effective approach to bringing this university’s teaching and learning into the 21 ^st^ century.

## Introduction

The massive change in communication and information technology in the past ten years raises questions about how, or even whether, we should harness this to teach our students. As teaching and research academics in a research-intensive university we are keen to engage students in “discipline knowledge” ( Breen, 1999), but what is an appropriate way to do that? Recording of lectures and ready availability of lecture notes and slides on-line is routine at most universities. This has led to heated debate about the role of presentation tools such as PowerPoint ( Horvath & Lodge, 2015; Sørensen, 2015).

The reduction in the number of students attending lectures in person has given impetus to calls for lectures to be replaced with other forms of teaching, or for recordings to be abolished. However, these may not be effective in addressing the real issue of student engagement, since the proportion of students downloading slides and listening to lectures is often less than 20% in any one week. Lectures did not disappear after the invention of the printing press, but they did evolve from the reading of texts to compilations of learned material. Current changes in digital technology indicate that there is clearly a need for lectures to evolve further.

The current student cohort, sometimes referred to as “Millennials”, is often accused of being self-centred and lazy ( Stein, 2013). This generalisation ignores the challenges these students face in juggling increasingly busy lives with the competing demands of work/life/study ( Abbott, 2013), and the increasing diversity in backgrounds, cultures, ages and life styles within the student cohort as a whole. As a result the current approach to study is more targeted and goal oriented. Students are used to sourcing information rapidly, with minimum financial and mental cost, and to be entertained in the process. Simple, well-organised websites such as Wikipedia are particularly useful for sourcing information in a targeted way. As educators, our role is to not just help students fulfil the minimum requirements to pass, but to inspire students to take control of their own learning, rather than just consume. Here we present a case study to serve as a model for organising and contextualising content and learning to better engage students, helping them develop effective learning strategies, and to show how attention to design principles can transform the student experience.

## Rationale: Designing for learning

Learning management systems are, too often, used as a file repository by busy academics that then gets rolled over from one year to the next, without change. We face the university-wide challenge of ensuring deep content that is both engaging and accessible. This can be addressed by creating interactive content that ensures students can readily access key information – for example, an interactive glossary lets students check their understanding of new keywords. The pedagogical challenge of clearly defining the purpose of learning and assessment activities is best addressed when it is clearly contextualised within unit objectives.

Learning design can be described as the “the complex process of planning, decision making, design, and creativity in [the] facilitation of student learning” ( Laurillard *et al.*, 2013). It is different from planning for learning that must consider institutional constraints such as timetabling, mode (face-to-face, online, blended), class sizes, and sociocultural backgrounds of students. When re-designing this unit, we wanted to take the opportunity to place the student at the centre the process. The optimal situation is one where the students are directed through learning activities designed to deconstruct the concepts and make the relationships between them transparent ( Laurillard, 2012).

**Figure 1.**
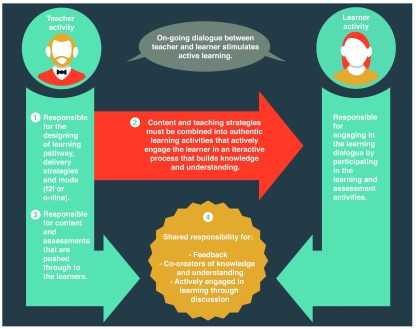
The Learning pathway, delivery strategies and shared responsibility in the learning environment. Based on Laurillard's conversation model reflecting teacher/student interaction (BTBL Bytes, 2015).

Our plan was to bring sound pedagogical theory of learning together with a smattering of instructional design to create a blended learning methodology that makes sense to busy academics in a university context. Dalziel (2009) refers to two determining factors to designing for learning: (1) the building of a learning pathway to sequence learning activities, and (2) the description and dissemination of practice. In this case study, we describe the steps and rationale taken in re-designing the learning pathway in a large undergraduate science unit, its impact on the students, and the plans for disseminating this model of good practice to other faculty members. Building a learning pathway requires a re-framing of the content and learning activities for the students and incorporates considerations of learning outcomes and resources that maximise the opportunities for learning through interaction with self (through reflection), and with peers as well as with the teacher (face-to-face and online channels). Thus the strategies for learning in the online environment can establish learning pathways that encourage students to explore and discover their own way through the content. ( Sims *et al.*, 2002). Figure 1 graphically represents Laurillard’s Conversation Framework with its emphasis on teacher-student interaction in the learning process and their shared responsibility when developing understanding. This interactivity is an essential component for the successful implementation of teaching and learning ( Sims *et al.*, 2002), and we suggest that all students will benefit from a course that is designed with the learner at its heart.

Students learn best when they are actively engaged and can construct their own knowledge ( Laurillard, 2012). Teachers and learners both play roles in this process (See Figure 1). It is the teacher’s responsibility to lower the barriers for learning by clearly outlining details of the assessments and the sequence of topics and related learning activities and ensuring that the content, learning activities and that assessments are aligned to the learning outcomes ( Gleadow *et al.*, 1993). Delivery strategies include pre-class, in-class and post-class teaching and learning activities that could include group project work, class presentations, excursions, guest speakers, and so on. How this learning takes place (e.g. face-to-face, blended, online, workplace or an internship) should be chosen on the basis of its effectiveness in the context. Students participate by engaging in these activities. This can be challenging and it is important to involve students in this process and to be clear about what is expected of them. Feedback is critical in the learning and teaching cycle if students are to improve and consolidate their learning. Students expect timely and detailed responses to their queries and qualitative feedback on their work ( Hannon *et al.*, 2002).

## Implementing learning design principles

### Getting started: rethinking the role of the learning management systems

**Figure 2.**
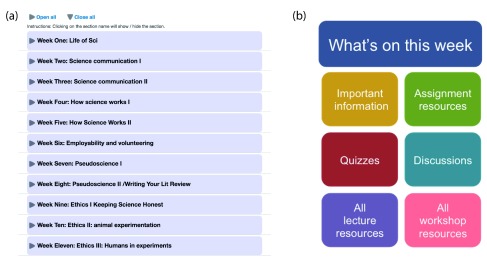
Landing page for students on the learning management systems. ( **a**) Previous layout with week-by-week arrangement, typical of many courses; ( **b**) Menu buttons and themes positioned on the front page.

The learning management system at our institution is Moodle, an open-source learning platform that is designed to give educators around the world a secure, integrated system through which to deliver learning. Academics have limited access to training in the effective use of Moodle and this combined with a busy teaching and research load means that we find, unsurprisingly, that Moodle is used in a very basic way. The default format is linear and without an understanding of learning theory to inform their decisions, academics use their online teaching space as a repository for their lecture slides and reading lists with a few forums thrown in. This was the situation with this core science unit when the learning designer started to work with the academic. Figure 2 is a very common Moodle page layout. This structure provides no clues for the learner as to where to find key materials such as assessments, lecture slides, resources and learning activities. These are hidden from the learner in this configuration.

The structure chosen for an online course should maximize the learning opportunities being presented: “Whatever medium is used for a text, its meaning is revealed through its structure” ( Laurillard, 2012) and in the online space, the structure needs to be the one that makes it easiest for the learners to navigate through the key points and supporting material. We addressed this issue over the first few months of the project, discussing the interplay between the face-to-face components of the course - the lectures and workshops - and the online space with reference to established principles of effective learning design and the underpinning pedagogy.

The first thing that a learning designer can be expected to address when embarking on a unit evaluation process is a critical analysis of the learning outcomes and their alignment to the delivery and assessment strategies. This process encompasses all factors that can impact on the learning environment including learning outcomes, curriculum, assessment, and teaching and learning activities ( Laurillard *et al.*, 2013). It is at this stage that inconsistencies can be identified and a process of remediation to address anomalies can be established. This was not the case with SCI2010. There was a rich, engaging face-to-face learning environment that was well established with a team of highly motivated tutors to support the academic in the delivery of a varied and stimulating learning environment that incorporated authentic assessment activities.

The assessments and learning activities were aligned, and the content was in a constant state of renewal; so what was left for a learning designer to do? What value could be added? Consequently, it was the online learning environment on which we focused our attention and we set about re-designing Moodle as a teaching and learning space rather than a filing system. We decided to re-build the online course to represent best practice in learning design and integrate sound pedagogic online facilitation protocols. These were: structure and organisation; aesthetic design; contextualisation; clear learning pathway; and online facilitation. Here’s how this worked in practice.

### Context: the student cohort and subject content

Our university is implementing a program for enhancing teaching that aims to ensure effectiveness through high quality design of learning outcomes and assessment regimes, multifaceted activities, and optimal delivery methods. The course chosen to spearhead a program for enhancing teaching across our university was *SCI2010 Scientific Practice and Communication*. The course deals with the nature and origins of science, ethical practice and science communication. This large, interdisciplinary unit is compulsory for all science students studying at the Melbourne and Malaysian campuses, or by distance education. There are approximately 1200 students (600 per semester) mostly in their second year of University and taking degrees in Physics, Biology, Chemistry, Biomedical sciences, Mathematics and Psychology. Students are, on average, 20.3 years old, with over one-third from homes where a language other than English is spoken (more at the international campus). A survey of graduates found that only one-fifth of students would have taken the subject if it was not compulsory, but in hindsight over two-thirds said they had learnt things not otherwise covered in their degree and that it should remain compulsory (unpublished data, The Faculty of Science, Monash University 2009). It is thus both challenging and rewarding to present the subject matter in a way that is intellectually engaging and relevant to students from a wide range of disciplines.

### Structure and organization – using headings, summaries, and a consistent framework

The course had always had a ‘Quick links’ section at the top of the page (as seen in Figure 2b). This guided the students to resources that they needed to access in a timely manner. These included *Assessments*, *Lecture resources*, *Quizzes*, and the highly rated, *What’s on this week?* that linked directly to the relevant page with the weekly learning activities. The buttons had been used for the past few years, and only required minimal modification to reflect the new navigation system. The unit Introduction (which was already in the Unit Guide) was brought into the online course front and centre, presenting this unit in context in the wider course and emphasising the learning outcomes as they applied in practice.

There was an obvious need to change the weekly headings to something that best described the topic and give the student more clues and support for their learning. As Al-Samarraie *et al.* (2013) propose, a well-designed structure underpinning the learning process will facilitate students’ understanding of the concepts leading to successful outcomes. The section headings were changed from *Week 1, Week 2* to topic names such as *Is science special? Can we afford self-deception? Can scientists be bad?* Each topic was presented with a consistent weekly structure to create expectations of learning activity. This structure included an introduction to the topic, the learning outcomes, and a pre-class activity to activate thinking for this week’s concept, learning activities – *Something to read, Something to do, Something to think about-* a series of questions for reflection, and the link to the lecture slides.

As we worked through this makeover, it became obvious that by contextualising the topics with more supporting information and activity, we were actually accentuating the major themes of the unit. It must be emphasised here that at no point did the content change, but rather the way that the learning resources and activities were presented changed the focus from a list of resources to a more thematically contextualised, learner-centred structure. This caused a re-think in the delivery and a shift in the paradigm to where the online space was truly connected to the face-to-face interaction.

### Aesthetic design – not just pretty

**Figure 3.**
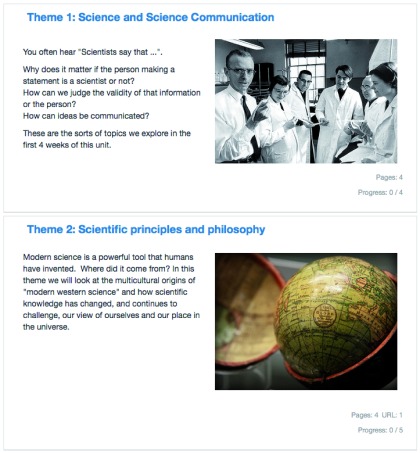
Example of front page of the learning management system, demonstrating how the content is organised into themes with arresting images. Pictures from Creative Commons. Image of scientists: Mars-discovery-district; Image of globe: Kotomi.

As a start, the design of the online space in Moodle was changed from collapsed topics to a more open setting. This different unit layout eschews the linear format, introduces images to guide the learner to different sections, whetting their appetite for further investigation. Each section in the new configuration has its own image and description to guide the students quickly to course material that they need ( Figure 3). This design and clear learning pathway was implemented to make obvious to the student the actions required to achieve specified learning outcomes. The learner is guided through scaffolded activities, discussions, opportunities for reflection, self-test quizzes, and extension activities if needed or desired. We believe that an altered learning landscape motivates the learners to engage with the prescribed materials and activities at a deeper level and reflectively participate in the learning experience. In doing this they are learning to become pro-active participants in their environment, actively reshaping their landscape to support on-going learning ( Goodyear, 2015).

**Figure 4.**
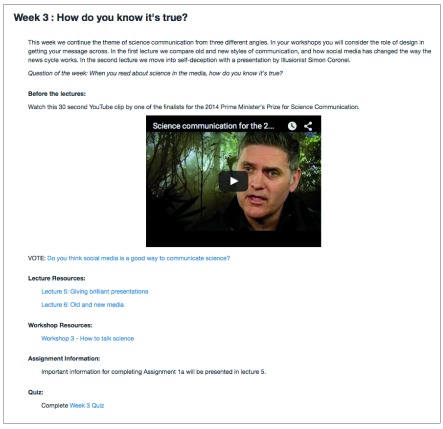
Science communication for the 21st Century. Question of the week - a Moodle polling activity. Students view a short video or image and are asked to participate in a poll. Video: Science communication for the 21st Century.

### Contextualising the learning

For us, contextualising the learning means adding value to the materials and activities presented online; interacting with the learners through the instructions and guidelines; and being present in that space with them, similar to that described by Laurillard (2012). In order to get to the underlying purpose of each activity, the learning designer (BM) prompted the teacher (RG) by asking: “If you were to present this video/activity/article to read in a face-to-face class, what would you say by way of introduction?” This forced the teacher to really think about the context and that then shaped the writing surrounding the learning activities so that the purpose was crystal clear, and would make sense to the students, forestalling questions such as: *Why am I doing this? What’s the point of this?*

**Figure 5.**
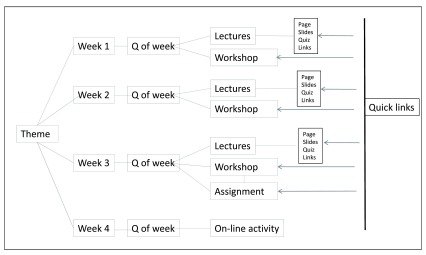
Schematic of layout of the page and logic connecting the various activities available through the learning management system.

The learning pathway was designed to guide the learner through clearly sign-posted themes and topics in order to help the students understand the progressive and cumulative building of knowledge; and to help them synthesise and apply the key concepts ( Figure 5). Students could, of course, access all the weekly resources through the quick links at the top of the page, and some did. However, our feedback and analytics of the hits per page suggest that most students took advantage of the learning opportunities presented in this format.

**Figure 6.**
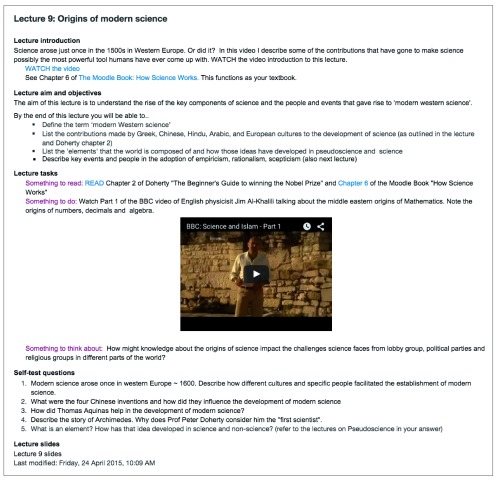
Example of a web page that accompanies one of the lectures. The same structure every week to create expectations of learning activity. The students are directed to the video BBC: Science and Islam - Part 1 in “Something to Do”.

A typical layout and introduction for each ‘lecture’ activity is shown in Figure 6. At the top there is a very short introduction, followed by a very short video and a Moodle Choice activity. This was designed to stimulate thinking on the topic, and by voting the students had to consider the issue and act, thus activating their thinking on the new concept and preparing them for the new learning. The next step for students is to access the *Lecture resources.* Again, the information was organised in a consistent format, designed to fit on a single screen of a computer, (see Figure 4) incorporating a short introduction, a list of learning outcomes, and a list of things to do post-lecture, called ‘ *Something to read’* (content and further reading), *‘Something to do’* (a related activity) *, ‘Something to think about’* (opportunity to reflect, extend and apply the learning.). The link to the lecture slides was at the bottom of the page.

In order for this blended model to be effective, all the tutors on the course were encouraged to participate in the online activities in private discussion forums (on-line and face-to-face) and bring those discussions into the classroom. The guided online activities were referred to and discussed in class, reinforcing to the students that the online space was valued as much as the face-to-face interaction. This seamless interaction in the online and face-to-face spaces highlighted to the students that the teachers were active in both spaces and the each mode was an essential element of the course delivery.

## Student evaluation and responses

There were 763 students enrolled in the unit: 633 at the Melbourne campus in Clayton, 109 at the campus in Kuala Lumpur, Malaysia (where students do the same program but have their own lecturers) and 21 students taking the unit by distance education. Data on student responses to the new layout and structure was collected in four ways. Firstly, we determined how many students were accessing the material and participating in the non-compulsory pre-lecture choice activities. Secondly, an online survey was conducted in Week 9 of the teaching program (see Supplementary material for wording of the questions). Thirdly, observations of attendance and engagement, and the type of questions that students were asking during the lead up to the final examinations were made.

### Participation and access

On average, 159 students took part in each pre-lecture poll, ranging from 415 in week 1 to 92 in the final week ( Table 1). Participation in the ten polls was completely optional, and attracted no marks. The weekly online revision quiz opened after the lecture and closed the evening before the lecture the following week. Students could make up to three attempts in this time. Students were rewarded with a ‘participating mark’ of 0.25% of the semester grade for attempting each quiz, but while they got feedback on their answers, no marks were given for getting the right answers. On average, there were over 1000 attempts at the quiz each week, which means that some students were attempting them multiple times.

**Table 1.** Number of students participating in the pre-lecture poll and revision quizzes in any particular week, and the overall average (± 1 standard error). There were 763 student enrolled in the unit across three campuses. Semesters run for 12 weeks (excluding the study period). There were no lectures in Weeks 4, 8 and 12.

Week	Poll question	Revision Quiz
1	415	1201
2	199	1351
3	178	1254
4	179	N/A
5	113	1006
6	123	852
7	131	957
8	N/A	N/A
9	69	966
10	91	924
11	92	619
12	N/A	952
**Average**	**159 ± 32**	**1009 ± 67**

### Student survey

There was the opportunity for students to provide open-ended responses (see Supplementary Data) as well as the ranking of specific aspects. Overall students were very positive about the changes:

*“Your Moodle site is awesome! I wish all our units were like that.”*
*“…By far my favourite moodle page of any subject.”*

**Figure 7.**
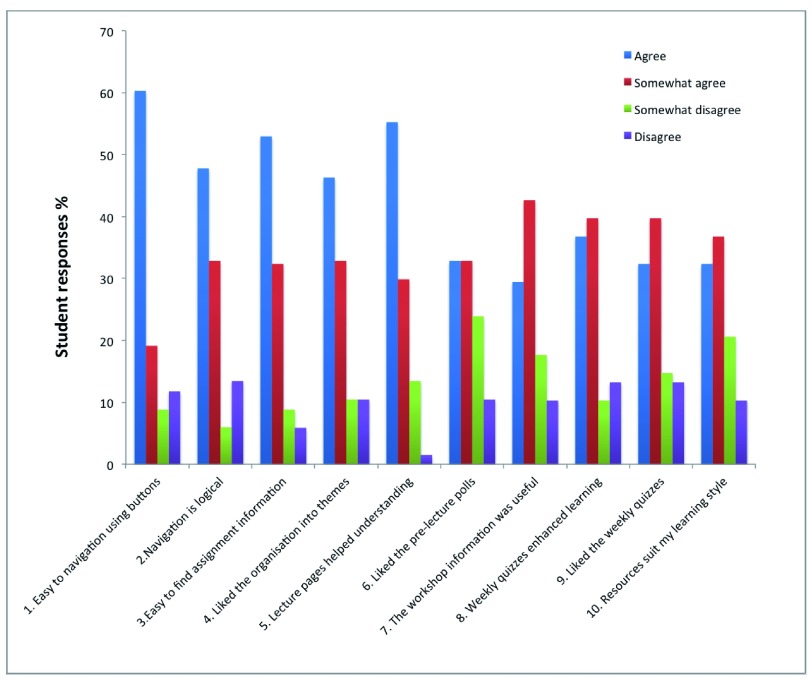
Percentage of students who Agreed, Somewhat agreed, Somewhat Disagreed or Disagreed with ten statements about various aspects of the revised structure of the learning management system (N=68). The full text of the questions is in Supplementary Data. The complete dataset is available as a.csv file.

Students were particularly positive to the questions about the navigation of the site, with 80.5% agreeing that the navigation was logical (Question 2), and the information easily accessible ( Figure 7). Students singled out the Quick Link buttons at the top of the page with 60.3% strongly agreeing that they liked being able to navigate the site using the buttons on the home page, and 55.2% strongly agreeing that is was easy to find information about the assignments. This was particularly rewarding, as a recurring complaint in student evaluations in past years was that it was hard to find out what was required for the assignments.

*“The start buttons made for excellent navigation…”*
*“It* [the quick link menu] *makes it easy to find the quizzes, information for workshops and information from lectures. Moodle isn’t always easy to navigate so this has definitely helped.”*

Of course, not everyone liked the new layout. The comment (below) highlighted one of the problems that we anticipated: if information is only accessible via the themes, then more clicking is required. It is good web design to be able to access the same material in several different ways, and this was incorporated into the design, but clearly this was not immediately obvious to everyone:

*“I didn’t like the organisation of the course content on Moodle. I much prefer the normal unit layout with everything on one page under different subheadings. It was often quite annoying having to click through many different pages in order to find information.”*

The major focus of the revisions was the lecture pages, while the workshop pages had only minimal information on them. Interestingly, this could be detected by the student responses. Over 55% of students strongly agreed and a further 30% somewhat agreed that “ *the lecture support pages helped me understand the lectures…*” but only 29% strongly agreed with the statement that *“*that “ *the workshop support pages helped me to get more out of the workshops”.* Other comments on the changes included:

*“Really liked the things to read, do and think about sections for every week. Really helped me keep on track with the subject and what is required.”*
*“Workshop resources should be linked to the lectures rather than having a separate tab.”*
*“I found the course content to flow logically from week to week.”*

The Moodle book was developed in response to students wanting more specific information; something particularly important in this course as there isn’t a textbook. Reluctant to move from good design and communication practice (few words, lots of images), I transferred and edited existing written materials into the on-line Moodle Book format. For example, feedback from 2014 included the following:

*“Suggest you make the slides more text rich? The way they are you have to listen to the whole lecture to work out what it is about”*

Not everyone worked out that this type of information was now available via the Moodle Book, as one student commented in the present survey:

*“please put more explanation and sentences in the slides instead of a bunch of images”*

As so often happens in education, students like what you’ve done and then want more of it. For example, the Moodle book is the first one of its kind in the Faculty of Science, but one student commented:

*“I like the moodle book however it should be supplied by a pdf.”*

Raw dataset for Gleadow *et al.*, 2015 ‘Design for learning – a case study of blended learning in a science unit’ An online survey was conducted in Week 9 of the teaching program to ascertain student satisfaction with the program. There was the opportunity for students to provide open-ended responses as well as the ranking of specific aspects. This dataset contains the results for the non-open ended portion of this survey. Click here for additional data file. Copyright: © 2015 Gleadow R et al. 2015 Data associated with the article are available under the terms of the Creative Commons Zero "No rights reserved" data waiver (CC0 1.0 Public domain dedication).

### Other qualitative assessments of the changes to the learning management system

In a typical semester lecture attendance can fall below 20% by the end of semester. Although we did not collate the data formally, counts of students at the end of the implementation program showed a shift closer to 25%. As expectations rise, we expect attendance to further improve. Why does this matter? Lectures should be the place where we inspire, direct and interact with students. By contextualising lectures, and making them part of a blended learning program, students should be better equipped to engage intellectually, and not be passive recipients of information.

Students in this course actively engage in Discussion forums during semester and in the lead up to the examinations. This semester there were far fewer questions asking for clarification about the objectives of each topic, and more questions about the application, reflecting deeper learning. For example, comments along the lines of:

“ *What are we supposed to know about the lectures on….?*”

Were replaced by comments such as:

*“Just wanted to clarify one of the examples on….given in the lecture...”*

## Logistics of implementing change

The learning designer (BM) and the teacher (RG) met for an hour every few weeks at first and then weekly as momentum gathered over a three-month period. At first, discussion was focused around the nature of learning and how best to facilitate that in an online environment. A teaching associate and education technologist (MH) was employed who would implement our ideas into the LMS. The learning designer modelled the design in a separate “trial” shell by importing the course’s artefacts and building samples of the Assessment block, the Choice activity, the contextualisation of activities and resources. MH implemented the design ideas into the LMS, incorporating design elements to simplify and enhance learner experience. RG then re-worked the content to suit the new design and wrote introductions and other supporting and instructional text to guide the learner through the material. We were able to access funding through the Faculty of Science to pay for approx. 50 hours of support for MH to build the course according to the specifications and source images for each section and videos for the pre-class activities and to build the topic quizzes (including the introduction of new styles of question), which proved to be very popular with students for revision.

We now have templates for other teaching coordinators to use to guide them through the process. Other units in the Faculty of Science have adopted this design and other faculties are also using a modified version of the unit template and branding it as their own. The topic and lecture templates are in the form of Word documents for those unfamiliar with working in the Moodle LMS and these templates have been developed as Pages in a course called “Model Moodle” for others at our institution to import into their own course and edit. With these resources, unit enhancements similar to our own can be implemented by the unit coordinator with some support and training, and indeed this is already happening. There was no multimedia magic, just looking at the LMS differently and seeing the possibilities. This makes it scalable and easy to replicate. The hard part is in the shift in mind-set to a more interactive and contextualised presentation of learning materials to activate deeper thinking and conceptual development.

## Discussion

The introduction of e-learning technology has been a game changer in education ( Mor & Craft, 2012). The LMS with its collaborative affordances now competes with the teacher for attention as many students tune into their lectures online rather than turning up in person. It falls to the academic – who is not usually trained as a teacher and is allocated little time or support to develop the newly required skill-set – to design a learning pathway that incorporates meaningful interactivity between the learner and the teacher; the learner and the online content and activities; and the learner and other learners. This focused interaction is critical to the success of the learner experience and will ultimately influence the efficacy of the learning environment. On the other hand, the use of digital technologies is taken for granted by students who expect that their lectures and assessments will be available to them online; but to the academic, who is time poor, anything beyond the basic online presence can be seen as window dressing. The onus is on academics to determine whether the increased workload and upskilling required to develop new digital resources is a good use of their time ( Sheey *et al.*, 2006). However, even busy academics will find the time to integrate methodologies if they are convinced that the change in practice will make a difference. Teachers’ use of learning technologies will increase if they are convinced that the pedagogy is sound, and if they are inspired and enthused enough to implement these changes into their teaching practice ( Macfarlan & Everett, 2010).

Management of cultural change requires simultaneous implementation from top-down and bottom-up ( Brown, 2014; Fullan, 1999; Patria, 2012). Top-down incentives include training, mentoring, showcasing, and research. Teaching and learning are high on the agenda of the senior management of our university and there is a concerted effort from all stakeholders to challenge the current *status quo* of teaching and effect a cultural shift with a move towards interactive rather than didactic teaching approach using innovative, effective and efficient online and face-to-face teaching modalities, providing the opportunity for deeper and relevant learning to be realised. (Better Teaching Better Learning vision, OVPLT, 2014). The provision of Education Designers can be a highly effective way of fostering innovative approaches to teaching and learning and guide academics towards the development of a pedagogy that incorporates digital resources. For a successful change process to be enacted Fullan & Stiegelbauer (1991) suggest that the likelihood of success will be higher when the individual's personal goal align with the organization's goal. It then falls to management to communicate the need for change and clearly articulate the support offered to manage this intended change.

Bottom-up incentives come if academics see that the process will lead to improved student outcomes that are simple and time-efficient. Academics are more likely to engage with cultural change if it is manageable, supported, and endorsed by their peers ( Patria, 2012). It takes the example of academics who act as change agents to implement this shift and undergo the rigors of detailed student feedback before others are prepared to follow. As the number of early adopters builds and the ideas build through and across campus the number of those ready to adopt new ideas and practices reaches a critical mass. The innovators need to be supported to share this practice with others ( McLaren & Kenny, 2015). This was the case with this science unit; the positive student feedback was a motivating force for other academics who are now interested in “unit enhancement”. Academics across faculties are keen to use ideas taken from our unit to enhance learning and testing in their own online learning space – technical and educational support in this regard is clearly in high demand. This development of consultation, support and modelling good practice involves the academics in “…a dynamic process that enacts participants [academics] to reflect about the values, processes and outcomes of an educational intervention.” ( Ghislandi & Raffaghelli, 2015). The outcome is the development of a model for an iterative process and a culture of reflective practice that encourages experimentation with new tools and pedagogical approaches in learning design. Our model is expected to be further refined, guided by evaluation of students’ motivation and learning outcomes.

The changes described here focused on structure and design, with only relatively minor modifications to the actual course content. Nevertheless, change needs to be carefully managed, so that those affected are brought along with it, and not alienated. The initial hesitance in implementing the changes proposed by the model described in this paper arose from the concern that if the course looked different to other courses, then it might be less acceptable to students. Indeed, the organisation had been converted from a Lectures/Workshops/Assignments layout to the weekly list in 2012 in order to be consistent with the majority of other teaching units. Students do not always appreciate change, possibly because it means they cannot generalise from one task to the next. They are mostly interested in the assessments and passing an exam. Vygotsky’s (1978) social learning theory means little to students whose workload is increased by a teacher’s exhaustive use of the collaborative affordances of the LMS. We spent a short time in each lecture referring to the LMS and talking about the outcome of the pre-week polls, however as only a minority of students attend lectures this was a bit like preaching to the converted. In order to help students during this time of transition it is going to be necessary to spend time explaining the rationale of this altered design, giving them the opportunity to reflect on their own learning ability and extending their understanding of the part they play in the teaching and learning process ( Ghislandi & Raffaghelli, 2015).

## Final reflections

The design we have described here used a consistent thread as a strategy within which we could create learning opportunities for a diverse group of students. The underlying principle was: *How can we help our learners to move from their current state of learning development to that “sweet spot” where what they know meets what they need to know.* We aimed to create an environment where the learning tasks were not so easy that the learners became bored and switched off, but were sufficiently challenged and motivated to work through difficult tasks with support from teachers and peers. We liken this to Vygotsky’s Zone of Proximal Development (1978) that describes the distance between the actual development of a learner as determined by independent problem solving ability and the potential development as evidenced through collaboration with others.

Such change is inevitable. Our learners can now readily access up-to-date information anywhere, anytime, and this requires us to adapt our methodology to meet increasingly complex challenges. As a consequence, we need to move “from distributors of knowledge to designers of learning experiences.” While it still falls to the teacher to manage content and assessment, a student-centred learning paradigm necessitates a collaborative learning environment where learners explore, enquire, analyse, and engage in authentic learning activities. There is, however, little support for teachers in developing skills in design for learning and a paucity of culture to nurture such practices.

Getting buy-in from other time-poor academics can be challenging. The pain is more in taking time to rethink what it is that you want to teach rather than the implementation. Making design explicit and shareable delivers consistency and makes implementation straightforward. The key lesson has been to set up in a step-wise manner with judicious use of time release so everything doesn’t have to be ready before the start of the teaching period. It is also possible to lower the hurdles by generating generic pages ready for the content to be added. Teaching academics should be encouraged to experiment with the technology available to them and, by reflective practice, work out what suits them. Institutions that support them through this process will help create a better learning environment for both lecturers and students.

## Data availability

The data referenced by this article are under copyright with the following copyright statement: Copyright: © 2015 Gleadow R et al.

Data associated with the article are available under the terms of the Creative Commons Zero "No rights reserved" data waiver (CC0 1.0 Public domain dedication). 

*F1000Research*: Dataset 1. Raw dataset for Gleadow *et al.*, 2015 'Design for learning – a case study of blended learning in a science unit', 10.5256/f1000research.7032.d101855

## Supplementary materials

**Student survey.**

We provided the opportunity in Week 9 for students to provide open-ended responses as well as the ranking of specific aspects of the program.
