# Supplementary material for: Design for learning – a case study of blended learning in a science unit
Source: F1000Res. 2015 Nov 16;4:898. Originally published 2015 Sep 24. [Version 2] doi: 10.12688/f1000research.7032.2 (PMC4648204; doi:10.12688/f1000research.7032.2)
Supplement: Supplementary file 2 [file f1000research-4-7909-s0001.tgz › 94f3f298-a549-4f06-aeb0-ba199cf4d3e7.docx]

### Supplementary Material

**Questions used to assess student responses to the reorganisation of the learning management system. Students were asked to rank each on the following scale:**

| Agree | Somewhat agree | Somewhat disagree | Disagree |
| --- | --- | --- | --- |

| Question |
| --- |
| 1. I liked being able to navigate the site using the buttons on the home page. |
|  |
| 2. Navigating course content is logical |
|  |
| 3. It is easy to find assignment information |
|  |
| 4. I like the organisation of the materials into themes |
|  |
| 5. The information on the lecture pages helped me to understand the lectures |
|  |
| 6. I liked the pre-lecture polls |
|  |
| 7. The information on the workshop pages helped me to get more out of the workshop. |
|  |
| 8. The weekly quizzes enhanced my learning |
|  |
| 9. I liked the weekly quizzes |
|  |
| 10. Overall, the learning materials and activities suit my learning style |
|  |
| Please comment on what you liked about the unit organisation in Moodle. |
