# Supplementary material for: Design for learning – a case study of blended learning in a science unit
Source: F1000Res. 2015 Nov 16;4:898. Originally published 2015 Sep 24. [Version 2] doi: 10.12688/f1000research.7032.2 (PMC4648204; doi:10.12688/f1000research.7032.2)
Supplement: Peer review history

# Peer review: Referee response for version 2

**DOI:** 10.5256/f1000research.7909.r11236

**Published:** 2015-11-19

**Authors:** Katharina Freund

**Affiliations:** 1 Australian National University, Canberra, ACT, Australia

The authors have appropriately addresses the revisions suggested by the reviewers, and the article is ready for indexation and dissemination.

I have read this submission. I believe that I have an appropriate level of expertise to confirm that it is of an acceptable scientific standard.

# Peer review: Referee response for version 1

**DOI:** 10.5256/f1000research.7570.r10478

**Published:** 2015-10-09

**Authors:** Katharina Freund

**Affiliations:** 1 Australian National University, Canberra, ACT, Australia

This article provides constructive and clear advice for learning designers and academics looking to re-design courses for blended delivery, and to make more effective use of the LMS (in this case, Moodle). I appreciated the accessible writing style, and would recommend this article to academics to assist in their teaching.

A few small comments:

1. I have some reservations about generalising student cohorts based on generational distinctions such as "Millenials", as this does not necessarily address the diversity of the student body. I do agree with your overall point - that it's easy to source information online - though.
2. There was a display error in Figure 7, where the numbers on the Y-axis of the graph displayed as signs (like #$).
3. I'm glad of the discussion on culture change as an important part of the process towards more effective use of Moodle, as I think this is one of the most important elements. I am reminded of the work on innovation diffusion in higher education - perhaps this might be relevant for inclusion in any future revisions. (See for example McLaren and Kenny 2015 in Australian Universities' Review).

I was curious as to the life of the project and workload involved in making these changes. How long did it take, and how many people worked on it? Obviously there was a positive effect on this course, and the new design was appreciated by students on the whole. How scalable was this initiative to more courses, and more institutions, and what sort of investment would be required?

Thank you for the invitation to peer review; I found the article insightful.

I have read this submission. I believe that I have an appropriate level of expertise to confirm that it is of an acceptable scientific standard.

# Peer review: Referee response for version 1

**DOI:** 10.5256/f1000research.7570.r10477

**Published:** 2015-10-07

**Authors:** Mary Williams

**Affiliations:** 1 American Society of Plant Biologists, Rockville, MD, USA

I enjoyed reading this article and recommend it for indexation.

I appreciate the authors’ understanding of today’s students, who are different from those of a generation ago particularly in their attitudes and expectations of their digital learning environment.  As this article indicates, course instructors and designers can be more effective in their instruction by leveraging today’s learners’ skills and abilities. There are two important messages for educators here.

1. **Use Moodle more effectively.** The authors present a before-and-after description of their redesign of a course Moodle page. It is common, as the authors say, for course organizers to use Moodle as an electronic filing system, but this usage does not particularly enhance a course. Rather, Moodle can be integrated into the course design to help students to navigate assignments and expectations. Furthermore, images and design elements are not difficult to incorporate into Moodle pages yet provide them with the contemporary feel our digital learners have come to expect. I would encourage the authors to disseminate their good ideas by sharing generic Moodle pages and template with others.
2. The expression “ **Something to read, something to do, something to think about**” provides a simple mnemonic device for instructors when designing a course and students when negotiating it that I think will enhance teaching and learning independently of the digital environment. As someone who has navigated several MOOCs, keeping track of assignments can be tricky; a schedule that requires certain tasks to be performed weekly will make the students’ job easier. I also like the way these three tasks span the spectrum of teaching methods: read this (student is relatively passive), do this (student must be more active), think about this (student must step back and grasp the big ideas that the first two tasks lead to).  This framework will also help instructors make sure that their reading lists and assignments are more than just busy work but are truly relevant to the week’s objectives.

 

Two small suggestions: I first read this as a printed PDF without hyperlinks, so I would have appreciated having all of the in-text references listed in the References section (e.g., Breen, 1999; Horvath & Loge, 2015 etc.).

Also, I found Figure 7 confusing. There seems to be a formatting problem on the Y axis. Furthermore, although the legend says “..Where 1 is a negative and 4 is a positive” the legend in the graph suggests that the leftmost column (blue) means Agree, which confuses me. Somehow this graph needs to be clarified.

I have read this submission. I believe that I have an appropriate level of expertise to confirm that it is of an acceptable scientific standard.
